# Nynaism

**Published:** 1896-10

**Authors:** 


### BOOK NOTICES.

NYNAISM. Nyna Publishing Company, Philadelphia. Price, $1.00.

This singular book is a small pamphlet of perhaps one hundred and twenty-five pages, having for its object the teaching of hygienic principles and the practice of them. According to this book the hygienic principles that should be practiced every day of one's life may be comprised under nine heads, the names of which all begin with the letter A—Administrativeness, Activeness, Agreeableness, Alimentation, Æration, Appeasement, Accoutrement, Ablution, and Actinism.

Although these names seem "far-fetched" and mysterious they are not so really, when properly understood, but are very comprehensive terms for certain perfectly obvious principles for keeping up the highest efficiency of the human mind and body.

Most people will read such a book as this, admire its assertions, tacitly agree with them, make empty resolutions to live up to the principles, lay the book aside, and then forget all about it. To prevent this, the reader is desired to enroll himself as a disciple of Nynaism—which is declared to be the name of a principle which comprises the foregoing nine subdivisions.

At the end of each chapter of the nine divisions of Nynaism is a blank page for the reader to record how much or how little he has followed out the teachings of the chapter. He also virtually agrees to spread these principles among his friends by giving circulars exhorting them also—not to *join*, for there is nothing to join—to become Nynaists by actually practicing these simple habits of regularity of life, cleanliness, and carefulness of person which mean so much for health.

Thus the subject is held ever before his mind, and his resolution stimulated to keep ever in the practice of health-giving modes of life. The whole plan is exceedingly ingenious, is a novel missionary method of inducing people who are habitually careless, and, it may be, ignorant of the laws of health to pay attention to these laws, and thus of widely benefiting humanity.

